# Sudden Severe Hypotension After Ultrasound Guided Femoral Nerve Block in a Fracture Neck Femur Patient: A Case Report

**DOI:** 10.1002/ccr3.70562

**Published:** 2025-06-12

**Authors:** Omar Faiz Al Mohamadamin, Bhavsar Rajesh Prabhakar, Thomas Stroem

**Affiliations:** ^1^ South Jutland Hospital Aabenraa Denmark; ^2^ Odense University Hospital Odense Denmark

**Keywords:** epidural spread, femoral block, hypotension, local anesthetic

## Abstract

Local anesthetic systemic toxicity or hematomas are speculated as reasons for hemodynamic collapse after femoral block, which can be avoided by ultrasound guidance. In case of severe hypotension despite standard precautions, local anesthetic spread towards epidural space can be suspected.

## 1 Introduction

Femoral nerve block (FNB) is useful for analgesia in fractures of neck and shaft of femur along with patellar injuries. It is also utilized alone or as part of a multi‐modal pain management plan [1, 2]. Apart from the common side effects of peripheral nerve blocks, such as nerve injury, allergic reaction, hematoma, infection, and inadequate blockage, femoral block may also show drug related side effects like local anesthetic systemic toxicity (LAST). Ropivacaine is the drug of choice as it shows better pharmacological safety when used in recommended dose range compared to other long‐acting drug such as Bupivacaine [3]. However, inadvertent intravascular injection may still induce a risk of LAST. We present a unique episode of hemodynamic collapse in an elderly patient with fracture neck femur despite performing ultrasound guided FNB under all precautions.

## 2 Case History

91‐year‐old female, known case of asymptomatic mild aortic stenosis, long‐standing hypertension, and mild chronic obstructive lung disease (COPD) brought to the emergency department after history of fall from cycle with pain in left groin and inability to stand. Patient was not taking any anticoagulation medicine. On arrival, patient was awake, oriented and hemodynamically stable. After initial assessment, fracture neck femur (FNF) was diagnosed. As patient must be operated within first 24 h, operation was scheduled for the next morning [4]. As a routine, all FNF receive FNB for pain relief. Under routine monitoring, that is, ECG, Pulse oximetry and noninvasive blood pressure and all aseptic precautions, FNB was administered using ultrasound guidance. Inj. Ropivacaine (0.5%) 20 mL was used as the local anesthetic. Inj. Clonidin 75 μg was added to prolong the analgesic effect. After 10 min, systolic blood pressure was found to be 60 mm of Hg. Heart rate was 84/min and oxygen saturation was 96% without oxygen. Immediately, Trendelenburg position was given. Fluid bolus of inj. ringer acetate was started. Inj. Ephedrine (10 mg) was administered to stabilize the BP.

### 2.1 Differential Diagnosis

At this point anaphylactic reaction, LAST and perivascular hematoma were most probable differential causes.

### 2.2 Investigations and Treatment

Along with thorough clinical evaluation, routine investigations such as hemogram, ECG, and cardiac enzymes, along with serum tryptase, were performed, which were normal [5]. Ultrasound of the groin revealed no hematoma. Focus‐assessed transthoracic echocardiography examination revealed no abnormalities in LV contractility; however, IVC collapsibility was increased, indicating hypovolemia.

Treatment: Before receiving reports of the biochemical reports, inj. Hydrocortisone and inj. Clemastin were administered to antagonize any possible anaphylactic reaction. In the clinical examination, the patient had no signs of CNS involvement seen in LAST, such as auditory changes, circumoral numbness, metallic taste, and agitation. Further, apart from hypotension, the signs of cardiac involvement seen in LAST, such as hypertension, tachycardia, bradycardia, and ventricular arrhythmias, were also absent. Despite the early resuscitative measures, the systolic BP remained under 90 mm of Hg with no neurological signs of LAST. The patient was shifted to high‐dependency unit. Infusion of phenylephrine was started to stabilize the BP. After 45 min, the BP was normalized, and phenylephrine infusion was tapered off. The patient was shifted to the ward and subsequently operated on the next day.

## 3 Discussion

Sudden severe blood pressure fall after femoral block in our case prompted several speculations such as LAST, hematoma or anaphylactic reaction. Absence of dyspnea, bronchospasm, skin rash, itching, and response to anti‐anaphylaxis treatment made it difficult to confirm the presence of allergic reaction. Further, absence of hematoma at the puncture site excluded hypotension due to blood loss. Therefore, LAST was speculated as the next probable explanation, despite the administration of a dose below the recommended maximum. However, the drug was delivered under ultrasound guidance with repeated aspirations to confirm extravascular needle placement, making inadvertent intravascular injection less likely. Furthermore, since central nervous system (CNS) symptoms typically precede cardiovascular toxicity even during intravenous infusion of local anesthetics (e.g., Ropivacaine or Bupivacaine at 10 mg/min), the absence of CNS signs in our case further argues against LAST as the underlying cause [6, 7].

Hence, cephalad spread of the local anesthetic toward the epidural space emerged as the next logical consideration.

The femoral nerve, which is the largest branch of the lumbar plexus, originates from the dorsal divisions of the L2–L4 ventral rami, travels through the abdomen, and enters the thigh beneath the fascia iliaca, inferior to the inguinal ligament [8]. An earlier case report on the 3‐in‐1 block, in which a catheter was placed high in the psoas compartment using Winnie's landmarks, described the entry point as just lateral to the artery near the accurately identified femoral nerve. The authors speculated that this approach could lead to epidural, subdural, or even subarachnoid anesthesia due to potential spread of the anesthetic [9, 10]. This observation supports the possibility of an anatomical pathway allowing communication between the femoral nerve sheath and the epidural space.

In our case, we speculate that the local anesthetic may have migrated from the femoral sheath into the epidural space, resulting in sympathetic blockade and subsequent hypotension.

Alternative techniques such as Fascia Iliaca plane block (FIPB) and pericapsular nerve group (PENG) block were investigated for per operative analgesia in similar situations.

The PENG block is a novel technique that targets the articular branches of the femoral, obturator, and accessory obturator nerves. It offers analgesia while sparing motor function, potentially allowing earlier postoperative mobilization. However, it is technically demanding and does not routinely cover the lateral femoral cutaneous nerve (LFCN), which may contribute to postoperative pain [11]. Further, awareness of anatomical variations and nearby structures such as the ureter is important to avoid injury [12]. The FIPB, on the other hand, has not shown encouraging results as compared to SHAM [13]. Recently, Spinal Phenol in Glycerol (SPING) block has been investigated as the analgesic strategy in nonoperative management of the proximal femur fractures [14]. However, it may not be the choice in perioperative settings due to its severe limitations [15].

## 4 Conclusion

Hypotension after FNB may be due to cephalad spread of local anesthetic drug toward epidural space.

## Author Contributions

**Bhavsar Rajesh Prabhakar:** conceptualization, data curation, formal analysis, investigation, software, supervision, writing – original draft, writing – review and editing. **Thomas Stroem:** conceptualization, data curation, formal analysis, investigation, methodology, resources, supervision, writing – original draft, writing – review and editing. **Omar Faiz Al Mohamadamin:** conceptualization, data curation, formal analysis, investigation, methodology, writing – original draft, writing – review and editing.

## Consent

During admission to the hospital, patient has given consent for all procedures and data collection. The authors have obtained written informed consent for publication from the patient.

## Data Availability

The data that support the findings of this study are available on request from the corresponding author. The data are not publicly available due to privacy or ethical restrictions.
